# Metabolomics and network pharmacology–guided analysis of TNF-α expression by *Argemone mexicana* (Linn) targeting NF-kB the signalling pathway in cancer cell lines

**DOI:** 10.3389/fonc.2024.1502819

**Published:** 2024-12-02

**Authors:** Sunanda Kulshrestha, Anjana Goel, Subhadip Banerjee, Rohit Sharma, Mohammad Rashid Khan, Kow-Tong Chen

**Affiliations:** ^1^ Department of Biotechnology, GLA University, Mathura, Uttar Pradesh, India; ^2^ School of Natural Products Studies, Jadavpur University, Kolkata, West Bengal, India; ^3^ Department of Rasa Shastra and Bhaishajya Kalpana, Faculty of Ayurveda, Institute of Medical Science, Banaras Hindu University, Varanasi, India; ^4^ Department of Pharmacology and Toxicology, College of Pharmacy, King Saud University, Riyadh, Saudi Arabia; ^5^ Department of Occupational Medicine, Tainan Municipal Hospital (managed by ShowChwan Medical Care Corporation), Tainan, Taiwan; ^6^ Department of Public Health, College of Medicine, National Cheng Kung University, Tainan, Taiwan

**Keywords:** anti-cancer, mexican poppy, colon cancer, medicinal plants, PI3/AKT pathway, skin cancer

## Abstract

**Introduction:**

Cancer has emerged as one of the leading causes of fatality all over the world. Phytoconstituents are being studied for their synergistic effects, which include disease prevention by altering molecular pathways and immunomodulation without side effects. The present experiment aims to explore the cancer preventive activities of *Argemone mexicana* Linn leaves extract in skin cancer cell lines (A431) and colon cancer cell lines (COLO 320DM)). In addition, TNF-α expression patterns and NF-kB signaling pathways have been examined.

**Methods:**

LC/MS study of *Argemone mexicana* Linn extracts in various solvents revealed anti-cancerous phytoconstituents. Network pharmacology analysis used Binding DB, STRING, DAVID, and KEGG for data mining to evaluate predicted compounds using functional annotation analysis. Cytoscape 3.2.1 created “neighbourhood approach” and networks. The MNTD of these extracts was tested on L929 fibroblasts. Skin cancer (A431) and colon cancer (COLO 320DM) cell lines were tested for IC50 inhibition. Evaluation of TNF-α and NF-kB expression in cell culture supernatants and homogenates reveals anti-cancerous effects.

**Results:**

LC-MS analysis of extracts predicted the presence of anticancer alkaloids Berberine, Atropine, Argemexicin, and Argemonin. In Network pharmacology analysis, enrichment was linked to the PI3-AKT pathway for both cancer types. MNTD was calculated at 1000μg/ml in L929. The ethanolic extract at 1000μg/ml significantly inhibited skin cancer cell proliferation by 67% and colon cancer cells by 75%. Ethanolic extract significantly reduced TNF-α expression in both cell lines (p<0.001), with the highest inhibition at 1000μg/ml. In TNF-α stimulated cell lines, 1000μg/ml ethanolic extract significantly reduced the regulation of the NF-kB pathway, which plays a role in cancer progression (p<0.001).

**Conclusion:**

*Argemone mexicana* Linn. known as ‘swarnkshiri’ in Ayurveda has been reported to be used by the traditional healers for the treatment of psoriasis and its anti-inflammatory and anti-cancerous properties, according to the Indian Medicinal Plant dictionary. In the experiment, the abatement in the expression of inflammatory cytokine TNF-α and inhibition of NF-kB transcription factor activation could be linked with the downregulation of cancer cell proliferation. The study revealed the anticancer activity of *Argemone mexicana* Linn in the cancer cell lines and paved a pathway for molecular approaches that could be explored more in *In vivo* studies.

## Graphical Abstract

Assessment of anti-cancerous activity of *Argemone mexicana* by its leaf extracts on skin and colon cancer cell lines using network pharmacology in conjunction with research into the regulatory patterns of TNF-α and NF-kB.

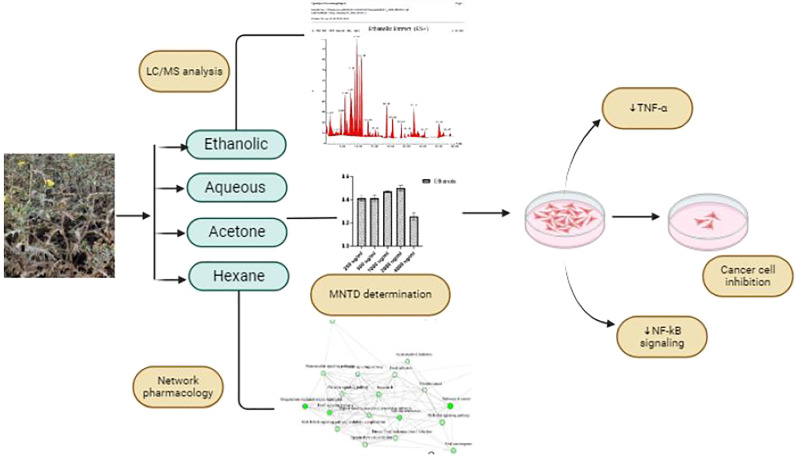

## Highlights

- *Argemone mexicana* Linn. is known for its anti-inflammatory properties.
- Ethnomedicinal use of the plant is employed to treat skin diseases like psoriasis.
- LC/MS analysis of extracts was followed by network pharmacology studies.
- 1,000 µg/ml of the ethanolic extract exhibited significant anticancer effect in cell lines.
- Downregulation in TNF-α and NF-kB was observed by ethanolic extract at the same dose.

## 1 Introduction

Cancer is prominent worldwide, leading to an increased burden on healthcare and the economy of any country (1). The number of cases of cancer is increasing at an exponential rate because of prolonged exposure to carcinogens, UV light, genetic factors, eating habits, and stagnant lifestyles, which lead to a suppressed immunity population and give a niche for the development of such disorders (2).

Skin cancer is one of the most prominent cancers in the world accounting for 2–3 million cases diagnosed in the world in the past 5 years. With 1,08,270 new cases and 4,420 deaths all over the world, making it the fifth most common cancer in the world, and in turn a burden on the economy of any country (1). Apart from this, with an estimated 106,590 new cases and 53,010 cancer deaths per year, colorectal cancer is the second most prevalent cancer-related cause of death. It accounts for 11% of all cancer diagnoses worldwide and is one of the tumours whose incidence is rising (1).

The basic therapy given to a diagnosed patient includes chemotherapy, radiotherapy, surgery, and oral drugs, but the situation does not seem to be in control (3). The drugs after a certain limit start lingering on with the patient’s life rather than curing and hindering the metastasis. Hence, in such a situation, some inspiration could be drawn from the ancient literature of Ayurveda to study and find some ways to cure the malignancy and interfere with the process of spreading and growing, which could in turn help to prepare an arsenal against cancer (4).

*Argemone mexicana* Linn, from family Papaveraceae, is a well-known toxic plant reported for possessing anti-inflammatory properties (5). Its authenticity and use as ethnomedicine have been cross-checked from The Plant List (http://www.theplantlist.org/) and MPNS (https://mpns.science.kew.org/) where the plant was found to be occurring 38 times in medicinal sources. It is called “swankshiri” in Ayurveda and is given to patients as a natural purgative in the process of Virechana Panchkarma (6). It has been used for treating psoriasis and other skin diseases by traditional healers and has also been patented by Arora et al. (7) for its effective healing in psoriasis. The plant has also been seen for its anti-cancerous properties by traditional healers (8). The herb is also quoted in Indian Medicinal Plants (9) where it is found to possess alkaloids helpful in treating skin infections, warts, and tumours. Willcox et al. (10) claimed a study to cure malaria in Mali; a prospective, dose-escalating, quasi-experimental clinical trial was carried out there with a traditional healer who used a decoction of *Argemone mexicana* Linn (10). Evidence from clinical or preclinical studies could be not retrieved related to its effect on cancers, but the present study will prove its preliminary evidence in *in vitro* cell lines, which could be expanded further to preclinical studies later.

Cancer is a disease that is caused by orchestrated stimulation of inflammatory pathways. The study of regulatory pathways playing a role in inflammation gives a better understating of the prognosis of the disease (11). Reactive oxygen species (ROS) derived from either inflammatory/immune cells or the mitochondria of epithelial cells act as the central endogenous carcinogens that drive cancer-promoting signalling pathways which include nuclear factor-kB (*NF-kB*), *STAT3*, *AKT*, and *COX-2* and are linked with different stages of cancer progression. This highlights the important role of such signalling pathways and involvement of transcriptional factors especially NF-kB which could be targeted in therapy for cancer. Rossi et al. (12) quoted the importance of TNF-α in cancers as it regulates some critical cellular processes such as cell survival and apoptosis, which are disrupted by TNF-α and its receptors (12). Xia et al. (13) and Tao et al. (14) reported the expression of inflammatory cytokines, NF-kB pathway stimulation, and other immunological events that leads to inflammation and eventually to cancer.

The prior literature review gave the research statement which helped us to draw the experiment where we could contemplate on the pivotal role of TNF-α and NF-kB regulation in the skin and colon cancer cell lines under the influence of *Argemone mexicana* (Linn) crude extract. To give supportive evidence to the study, the network pharmacology approach has also been utilized. With the help of network pharmacology approach, we can shed light on the way forward for the modernization of traditional medicine research and development through network pharmacology analysis, which also allows us to forecast and analyse the likelihood of drug side effects and identify new targets of drug effects (15).

## 2 Materials and methods

### 2.1 Chemicals

Chemicals including MEM media (minimum essential medium), DMEM (Dulbecco’s modified Eagle’s medium), FBS (foetal bovine serum), and antibiotics (penicillin–streptomycin) were purchased from Sigma-Aldrich, USA. The standard chemotherapeutic drugs dacarbazine (Da) and doxorubicin (Do) were purchased from Pfizer, USA. Plasticware including culture plates and flasks was purchased from NEST, China. The cell lines of skin cancer cells (A431), colon cancer cells (COLO 320DM), and L929 (Fibroblast) were procured from NCCS, Pune. The LC-MS analysis was done from SAIF, CDRI, Lucknow. The TNF-α kit (Catalogue No.: ELK1190) and NF-kB kit (Catalogue No: ELK1387) were purchased from ELK Biotechnology.

### 2.2 Sample collection and authentication

The leaves of *Argemone mexicana* Linn were collected from local areas and villages surrounding GLA University, Mathura, during February and March (coordinates: 27.49°N and 77.69°E). The leaves were authenticated from CIMAP (Central Institute of Medicinal and Aromatic Plants), Lucknow, and the specimen sample was deposited with Voucher no. CIMAP/Bot-pharm/2021/09.

### 2.3 Preparation of extracts

The extracts were prepared by a hot extraction method using the Soxhlet apparatus (16). The ethanolic (Eth), aqueous (Aq), acetone (Ac), and hexane (Hex) extracts were prepared and concentrated using a rotary vacuum evaporator (Yamato Scientific Co., Japan).

### 2.4 LC/MS analysis

#### 2.4.1 LC and its parameters

The LC-MS analysis was performed on Acquity Ultra Performance Liquid Chromatography equipped with a Xevo TQD interfaced *via* an ESI source (Waters Co., Milford). The compounds were separated on a Thermo Scientific™ Accucore C-18, 150 × 2.1 mm, 2.6 µm reverse-phase column at a constant flow rate of 0.25 mL/min. The temperatures of the applied column and the auto-sampler were 35 ± 5°C and 25 ± 5°C, respectively. The mobile phase consisted of three solvents: acetonitrile (A), 0.1% formic acid buffer which was prepared in 95:5 v/v water/acetonitrile (B) using a multi-step gradient (**Supplementary Table S1**), and a sample injection volume of 2 μl. 1 mg of extracts was dissolved with 1 ml of methanol (LC/MS grade) and diluted to the concentration of 1 µl/ml. Then, the sample was filtered through a 0.22-µm PTFE membrane syringe filter before chromatographic analysis. The injection volume was 1 µl. The gas temperature was 350°C, and the gas flow was 13 L/min. Full-scan mass spectra in the range m/z 100–1,000 amu were acquired in positive and negative ion modes. The nebulizer was 45 psig. The analysis was performed by using Agilent MassHunter B.08.00 software (Qualitative Navigator, Qualitative Workflows) and the PCDL database (TCM database, phenolic acid, and PubChem chemical database). Peak identification was compared with the retention time, mass spectra, and fragmentation patterns with reference compounds from the library where accuracy was error less than 5 ppm and MS/MS fragment matching for each compound (17). The Mass Profiler Professional was used to perform chemometric differential MS data analysis to derive the relationship between the extract and their bioactivity. For qualitative analysis, full-scan data in both ES ± ion modes with a mass range of *m/z* 150–2,000 are summarized in **Supplementary Table S2** of the **Supplementary File**. The LC-MS data acquisition was done using MassLynx software version 4.1.

#### 2.4.2 Mass spectrometry parameters

For qualitative analysis, full-scan data in both ES ± ion modes in a mass range of *m/z* 150–2,000 are summarized in **Supplementary Table S2** of the **Supplementary File**. The LC-MS data acquisition was done using MassLynx software version 4.1.

### 2.5 Target search, protein–protein interaction, and functional association network analysis

The compounds were mined extensively for target prediction by SwissTargetPrediction (threshold >0.1). The target ID identified as a human target with accurate UniProtKB/ID was retrieved. STRING 3.0 was used to analyse protein–protein interaction (PPI) analysis. PPI networks, which are undirected graphs with proteins as nodes and known interactions between the linking proteins as edges, were analysed to exhibit them. To comprehend the organization and localization, additional topological and module analysis was conducted to identify the main hub proteins, their connections, and collective activities. For additional hub and module analysis, a minimal interaction network was chosen. The hub proteins are a representation of their essential signalling pathway roles. KEGG is used to analyse the protein’s functional importance. Cytoscape was used to create the networks (18).

### 2.6 Cell culture

L929, A431, and COLO 320DM cell lines were cultured and maintained in their defined medium as per the instruction from the cell culture repository, NCCS, Pune. The fibroblast cell line (L929) was used to determine the maximum non-toxic dose, and the skin (A431) and colon (COLO 320DM) cancer cell lines were used to study the cytotoxicity of the extracts with different non-toxic doses.

### 2.7 Determination of maximum non-toxic dose in fibroblast cell lines (L929)

200 µl of 2 × 10^6^ fibroblast cells/ml was seeded in a 96-well cultured plate using DMEM supplemented with 10% FBS and 1% antibiotics at 37°C with 5% CO_2_ along with different concentrations 100 µg/ml, 250 µg/ml, 500 µg/ml, 1,000 µg/ml, and 2,000 µg/ml of the *Argemone mexicana* leaf Linn extracts (Eth, Aq, Ac, Hex). The cells were seeded in triplicates. The maximum non-toxic dose (MNTD) was determined by using MTT proliferation assay after 72 h of culture. The optical density was measured at 570 nm.

### 2.8 Determination of anti-cancerous activity against cancer cell lines

200 µl of 4 × 10^4^ cells/ml from A431 and COLO 320DM was seeded in each well in modified MEM and RPMI 1640 media, respectively. The cells were incubated for 72 h with the non-toxic concentrations of the extracts *Argemone mexicana* Linn from 100 µg/ml to 1,000 µg/ml. Dacarbazine (DA) and doxorubicin (DO), standard chemotherapeutic drugs, were used as positive control in different concentrations 20 µg/ml, 40 µg/ml, 60 µg/ml, and 80 µg/ml. The percentage inhibition of cells was calculated by MTT assay after 72 h. The IC 50 value was calculated, and statistical analysis was done using GraphPad Prisma version 8.

### 2.9 Determination of TNF-α regulation in the cancer cell lines

A431 and COLO 320DM were cultured in 96-well plates as given above with different concentrations of *Argemone mexicana* Linn extract and standard chemotherapeutic drug. The culture supernatant was collected after 48 h. The concentration of TNF-α was estimated using an ELISA kit as per the manufacturer’s instruction.

### 2.10 Determination of NF-kB regulation in the cancer cell lines

1 ml of 4 × 10^4^ cells of A431 and COLO 320DM was cultured in 1-cm^2^ culture plates along with different concentrations of *Argemone mexicana* Linn extracts. The cells were cultured and left unstimulated as well as stimulated with 20 pg/ml of TNF-α (19) as per the protocol by Ernst et al. (20). After 40 min, the supernatant was discarded and the cells were collected with 0.25% trypsin and lysed with 1 M lysis buffer containing 10 mM tris HCl, 1 mm EDTA, 0.01% sodium deoxycholate, 140 mm NaCl, and 1× PMSF (21). The cells were further sonicated for 10 min for complete lysis. The cell homogenate was then centrifuged for 10 min at 1,500 rpm. The supernatant was collected, and the p65 transcription factor was estimated using ELISA kit according to its manufacturer protocol.

## 3 Results

### 3.1 LC/MS analysis

**Figure 1:**
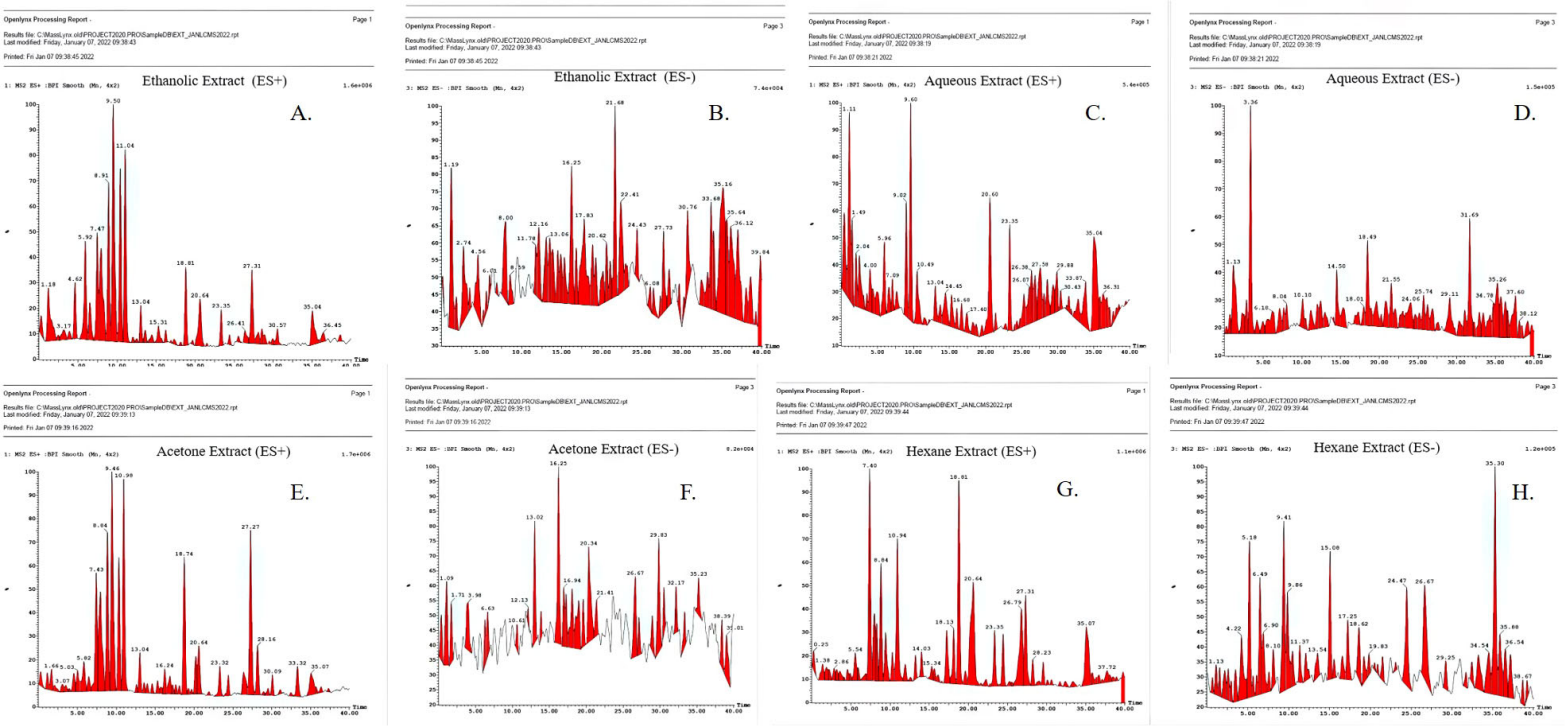
LC/MS spectrum of *Argemone mexicana* Linn leaf extract **(A-H)**. **(A)** Ethanolic extract (ES+). **(B)** Ethanolic extract (ES−). **(C)** Aqueous extract (ES+). **(D)** Aqueous extract (ES−). **(E)** Acetone extract (ES+). **(F)** Acetone extracts (ES−). **(G)** Hexane extract (ES−). **(H)** Hexane extract (ES−).

The mass spectra of the tested extracts are shown in **Figure 1** and predicted compounds in **Table 1**. Anti-cancerous compounds like berberine (mw: 335 da), octadecanoic acid (mw: 402 da), protomexicine (mw: 353 da), jatrorrhizine (mw: 337 da), oleandrigenin-ß-D-digi (neritaloside) (mw: 592 da) were predicted in ES+ and ES− spectra of different extracts. Detailed lists of the compounds with their predicted molecular weights are given in the supplementary file (**Supplementary Tables S3.1–S3.4**) with their biological activities. The spectrum analysis precited the presence of compounds having noted anti-cancerous activity from the literature.

**Table 1:** LC/MS-predicted compounds with their calculated molecular weights in different extracts of *Argemone mexicana* Linn.

Name of the compounds and with their molecular weights in Daltons (Da)				
S. no.	Aqueous	Ethanolic	Acetone	Hexane
1.	Protomexicine (353)	Oleic acid/octadecenoic acid (325)	Reticuline (339)	Linoleic acid (380)
2.	Allocryptopine (369)	Pentadecyne (322)	Argenaxine (196)	Arachidic acid (321)
3.	13-Oxoprotopine (278)	Argenaxine (196)	Protomexicine (353)	Magnoflorine (341)
4.	Octadecanoic acid (402)	Protomexicine (353)	Dihydrochelerythrine/allocryptopine (369)	Argenaxine (196)
5.		Allocryptopine (369)	Jatrorrhizine (337)	Protomexicine (353)
6.		Jatrorrhizine (337)	Berberine (335)	Allocryptopine (369)
7.		Oxyberberine (353)	Pancorine (333)	Berberine (335)
8.		Berberine (335)	Oleandrigenin-ß-D-digi (neritaloside) (592)	Argemonic acid (276)
9.		Chelerythrine (345)		Pancorin (333)
10.		Pancorine (333)		13-oxoprotopine (248)
11.		Dehydrocorydalmine (338)		Oleandrigenin-ß-D-digi (Neritaloside) (592)
12.		13-oxoprotopine/linoleic acid (278)		
13.		Oleandrigenin-ß-D-digi (neritaloside) (592)		

### 3.2 Compound–target–protein interaction network enrichment analysis

**Figure 2:**
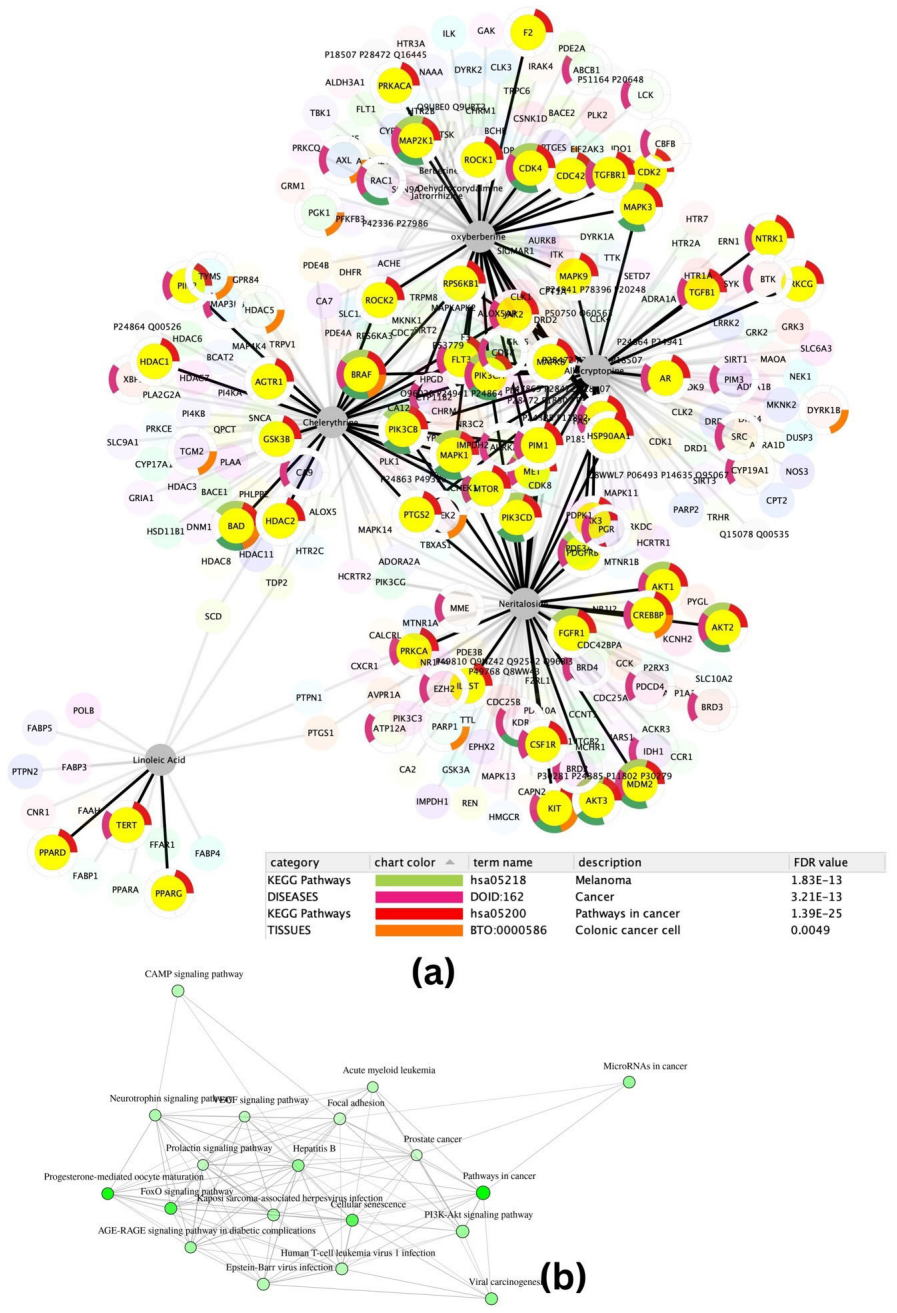
**(A)** Compound target–protein–protein interaction functional enrichment network of *A. mexicana*. **(B)** Pathway enrichment network.

The targets searched intersecting with the disease interacting space shown above (see **Figure 2**) were searched for crucial protein interactions in the STRING interactome. The protein–protein interaction network had 290 nodes and 434 edges highlighted to show enrichment for melanoma (p-value 1.83E−13) involving MAPK1, CDK4, MDM2, PDGFRB, MAPK3, PIK3CA, MAP2K1, MET, PIK3CD, AKT2, BAD, FGFR1, BRAF, AKT1, AKT3, PIK3CB; pathways in cancer (p-value 1.39E−25) involving MAPK1, TGFB1, RPS6KB1, FLT3, CDK4, MDM2, PDGFRB, CREBBP, MAPK3, PRKCG, PIK3CA, CDK2, CSF1R, PPARG, KIT, MAP2K1, F2, TERT, PRKACA, PPARD, MET, ROCK2, GSK3B, HSP90AA1, MTOR, PTGS2, ABL1, PIM1, HDAC1, AR, PIM2, PIK3CD, IL6ST, JAK2, AKT2, BAD, MAPK8, ROCK1, FGFR1, MAPK9, AGTR1, PRKCA, BRAF, HDAC2, NTRK1, JAK3, TGFBR1, AKT1, CDC42, AKT3, and PIK3CB. This network was then used to elucidate significant functional annotations further. The target functional enrichments are represented in a pie, where the pie is coloured according to the most significant functional terms which are regulated by melanoma represented by the green colour, cancer represented by red, pathways in carcinoma represented by purple, and colon cancer cells represented by orange (**Figure 2A**). Important targets related to skin and colon cancer are indicated by the yellow colour.

The compounds in allocryptopine, oxy-berberine, chelerythrine, and neritaloside all show a connection to the metabolic process. The PPI pathways study revealed melanoma (FDR: 1.823E−13), cancer (FDR: 3.121E−13), and colonic cancer (0.0049) in terms of enrichment, indicating that the active compounds are more likely to involve targeted pathways in melanoma than colon cancer. Additional research intends to investigate these links to their potential role in cancer.

**Figure 3:**
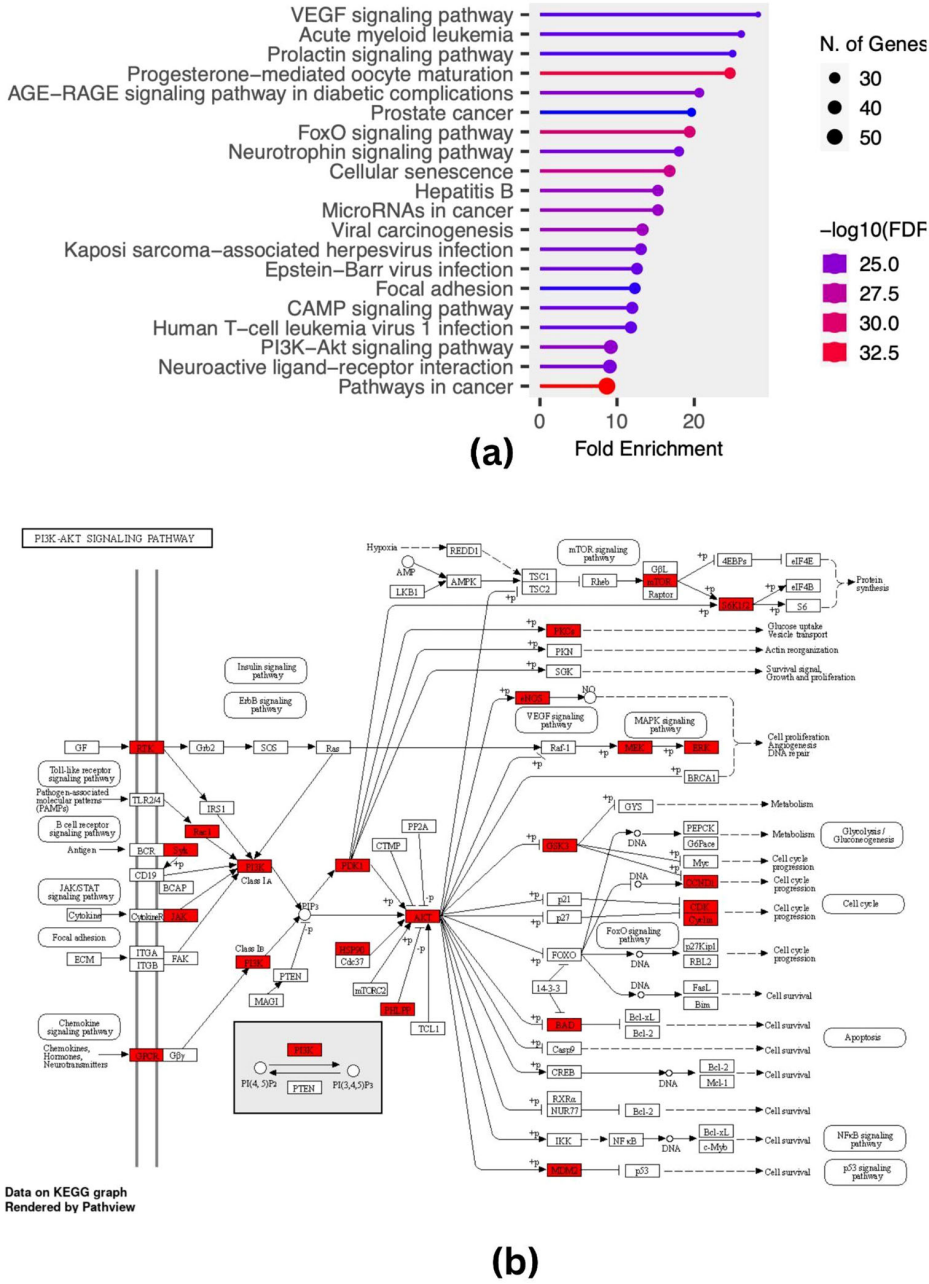
**(A)** Pathway enrichment chart **(B)** PI3K-Akt signalling pathway.

The network analysis of the molecules shows their importance in the promotion of cancer in colon and skin cancer cell lines. **Figure 2B** shows the enriched network clustered based on enrichment scores of pathway terms which were used in the network of pathway and process enrichment analyses. The distance between the targets is proportional to their involvement in the targeted pathways about their involvement in the enriched terms which were clustered according to their kappa scores with a similarity >0.3, and nodes reflecting significant p-values from each of the top clusters are connected by edges. We find a high correlation between microRNAs in cancer and the PI3K-Akt pathway. The PI3K-Akt signalling pathway was determined to have the highest likelihood shown in **Figure 3A**, which displays a comparative analysis and ranking of enriched pathways. This pathway is the best-ranked one, and it has 40 target genes that have a relatively small fold enrichment distance; these genes may be involved in skin and colon cancers. The PI3K-Akt signalling pathway as shown in **Figure 3B** is the most crucial one that has been highlighted.

The network pharmacology study found that the phytochemicals from *Argemone mexicana* have an effect on PI3K-Akt. It has been shown in multiple studies that NF-kB is involved in the PI3K-Akt signalling pathway. According to Tewari et al. (22), NF-kB is situated downstream of AKT, even though it is not directly involved in the main PI3/AKT pathway. Activation of AKT is essential for various tumorigenic NF-kB activities, which is tried to tap along with TNF-α expression. In diseases like skin cancer and colon cancer, where an overabundance of TNF-α production is a factor in disease progression, this study in expression is important to be noted.

### 3.3 Determination of MNTD in fibroblast cells (L929)

**Figure 4:**
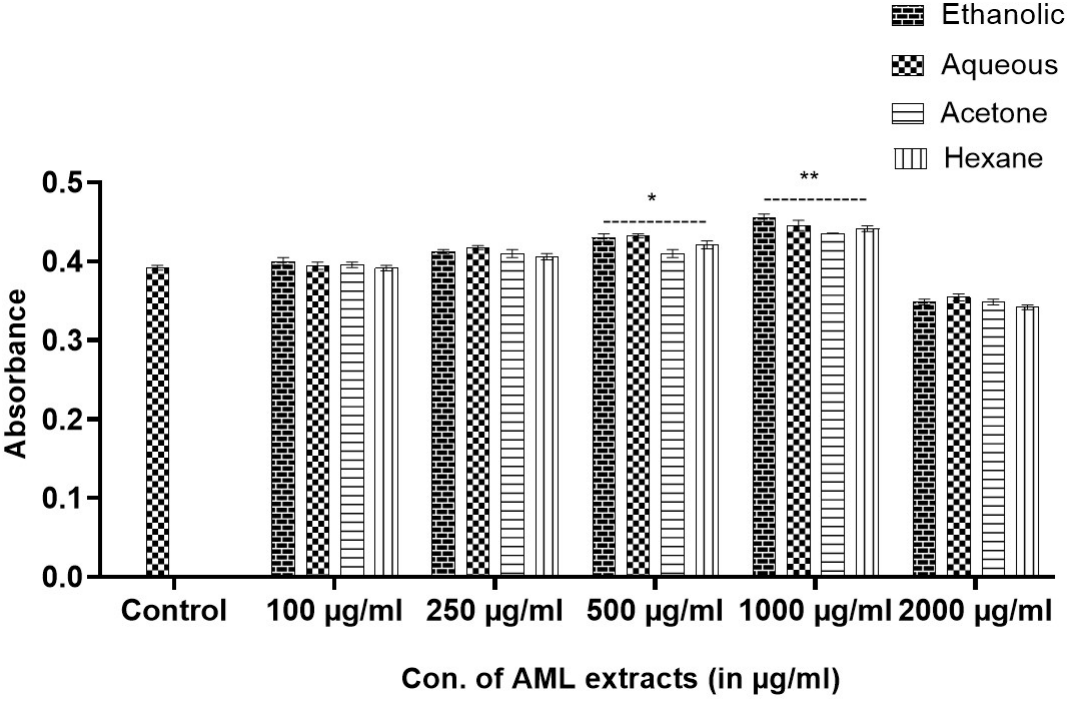
Determination of non-toxic doses of *Argemone mexicana* Linn extracts by MTT cell viability assay on L929 fibroblast cells. The extracts up to 1,000 µg/ml were found to be non-toxic, and a dose of 2,000 µg/ml was toxic to the cells.

After 72 h, MTT dye analysis revealed that the *Argemone mexicana* extract at 1,000 µg/ml was non-toxic and promoted fibroblast cell growth. The proliferation rate was 22.5% with Eth, 12.34% with Aq, 15.96% with Ac, and 11.54% with Hex extracts compared with control. The 2,000-µg/ml concentration was toxic, resulting in a −35% reduction in proliferation compared with the control (see **Figure 4**).

### 3.4 Determination of anti-cancerous activity against the skin cancer cell line (A-431) and colon cancer cell line (COLO 320DM)

**Figure 5:**
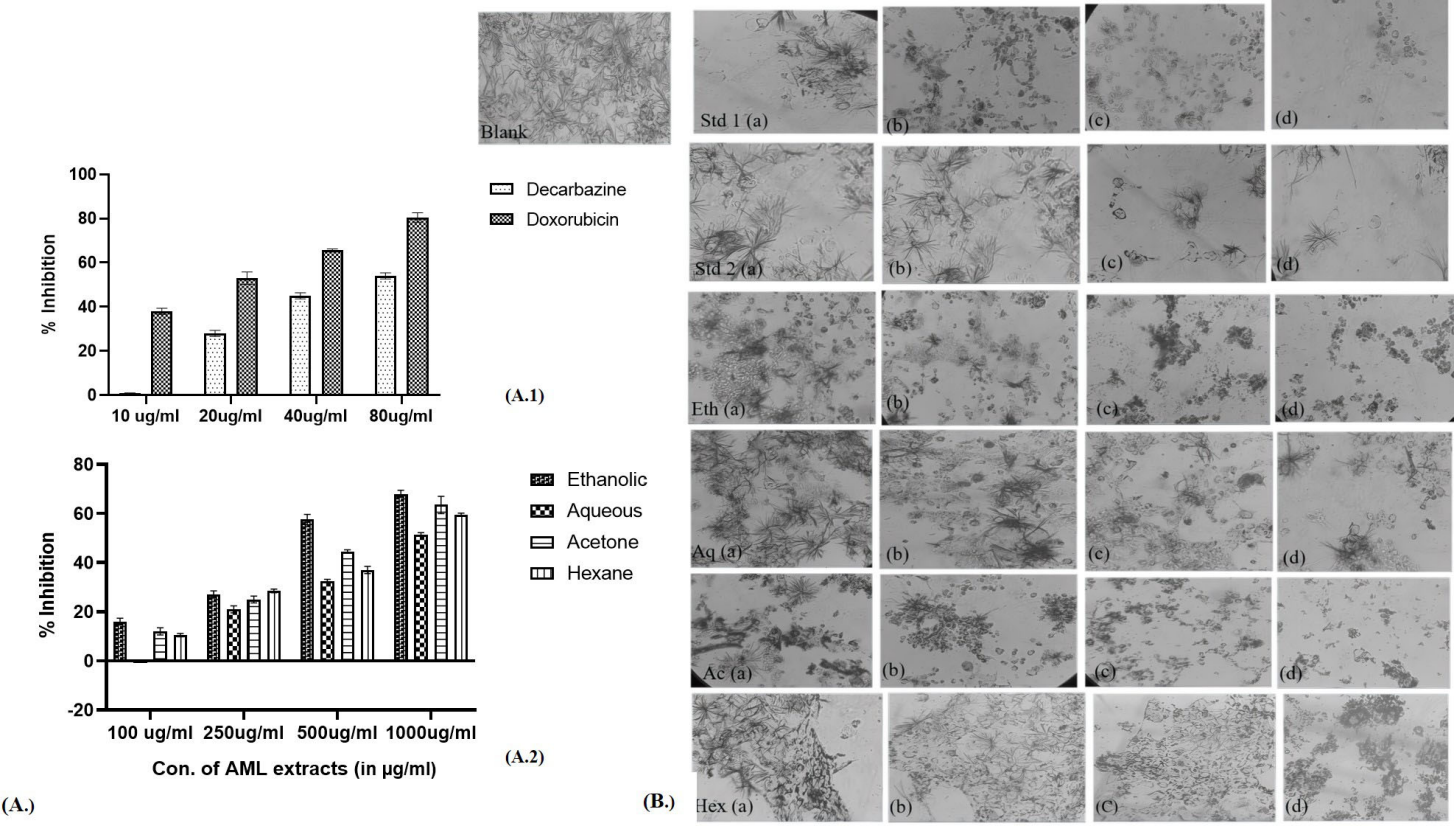
Percentage inhibition in the skin cancer cell line in the presence of different extracts of *Argemone mexicana* Linn leaves. **(A)** 1: percentage inhibition of skin cancer cells by doxorubicin and dacarbazine at 80 µg/ml. **(A)** (2.): depicts the inhibition to be reduced at 1,000 µg/ml by the ethanolic extract. **(B)**: Std 1 (a.) has been treated with 20 µg/ml, (b.) 40 µg/ml, (c.) 60 µg/ml, and (d.) 80 µg/ml of chemotherapeutic drug doxorubicin and the same dose has been administered in Std 2 (a-d) of dacarbazine. Eth (a-d) has been treated with 100 µg/ml, 250 µg/ml, 500 µg/ml, and 1,000 µg/ml of the ethanolic extract. The same doses have been given for Aq (a-d), Ac (a-d), and Hex (a-d). The cytotoxic effects become apparent when the number of cells decreases following the MTT assay.

**Figure 6:**
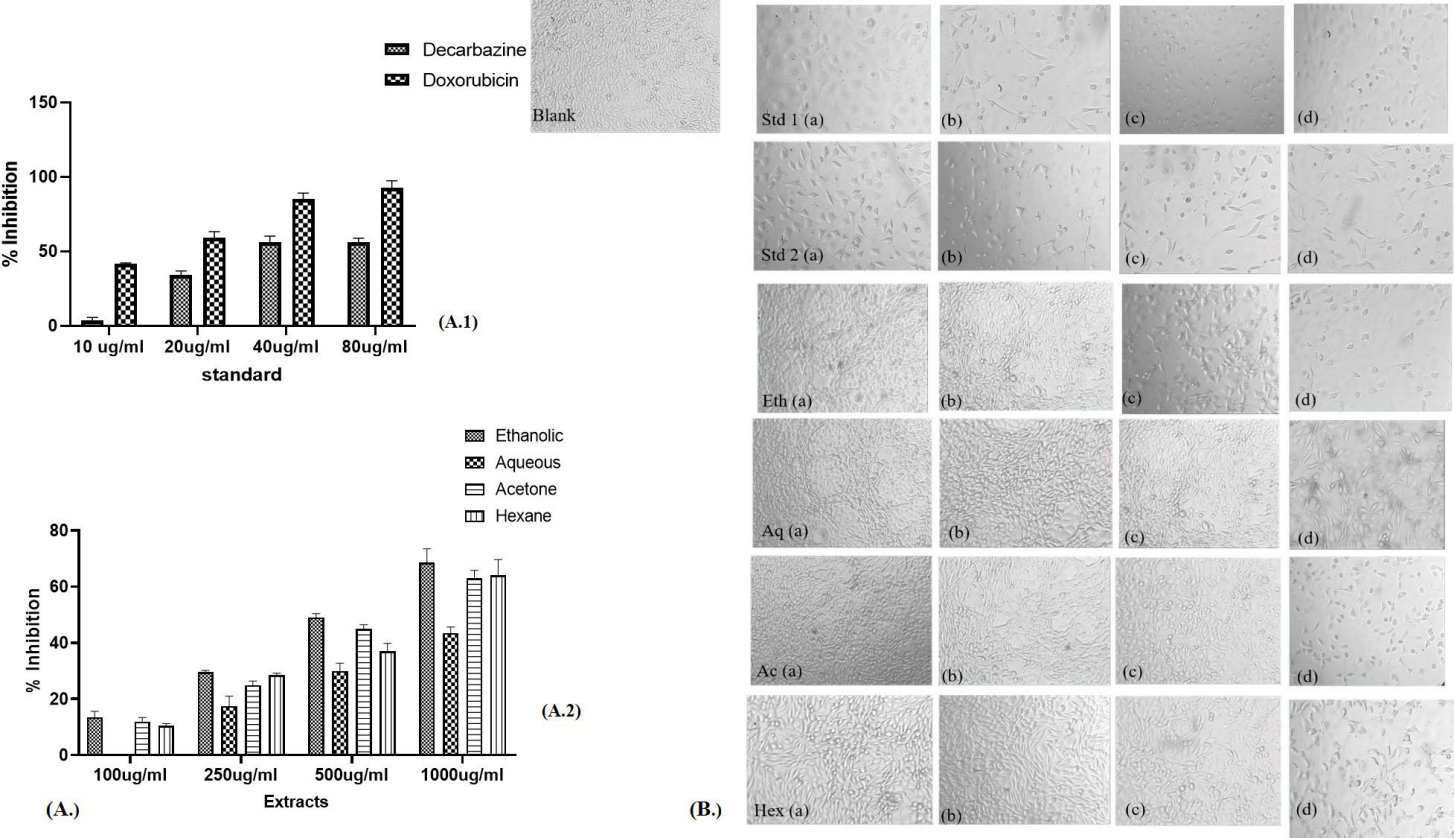
Percentage inhibition in colon cancer cell line in the presence of different extracts of *Argemone mexicana* Linn leaves. **(A)** 1: percentage inhibition of colon cancer cells by dacarbazine and doxorubicin at 80 µl/ml. **(A)** 2: The inhibition can be seen to reduce at 1,000 µg/ml by the ethanolic extract. **(B)** The doses Std 1 (a-d.), Std 2 (a-d), Eth (a-d), Aq (a-d), Ac (a-d), and Hex (a-d) have been given the same as skin cancer.


The cytotoxic effect of different extracts of leaves *Argemone mexicana* Linn was observed in cancer cell lines in the form of inhibition of proliferation, which is shown in **Figure 5** (skin cancer) and **Figure 6** (colon cancer). The significant differences were observed in form of percentage of inhibition calculated using the formula.

(Abs con−Abs sample)/(Abs con)×100=%Inhibition Activity

The extracts showed dose-dependent effectiveness, with ethanolic extract at 1,000 µg/ml inhibiting cell growth by up to 68.7%. In skin cancer cells, the IC50 for standard chemotherapeutic drugs was 73.75 µg/ml for dacarbazine and 19.63 µg/ml for doxorubicin. The IC50 values for AML extracts were 497.26 µg/ml for ethanol, 923.97 µg/ml for aqueous, 547.32 µg/ml for acetone, and 610.3 µg/ml for hexane. **Figure 5** presents the pictures of skin cancer cells treated with AML extracts. **Table 2** presents the IC50 values for respective AML extracts for A-431 and COLO 320 DM cell lines.

**Table 2:** Comparative IC50 values of standard chemotherapeutic drugs and AML extracts on skin and colon cancer cell lines.

	IC 50 for A-431 (in µg/ml)	IC 50 for COLO 320DM (in µg/ml)
Standard chemotherapeutic drug		
Dacarbazine	73.75	35.23
Doxorubicin	19.63	15.89
AML extracts		
Ethanolic	497.26	502.35
Aqueous	923.97	–
Acetone	547.32	623.36
Hexane	610.3	821.13

### 3.5 Determination of TNF-α concentration in skin and colon cancer cell lines

**Figure 7:**
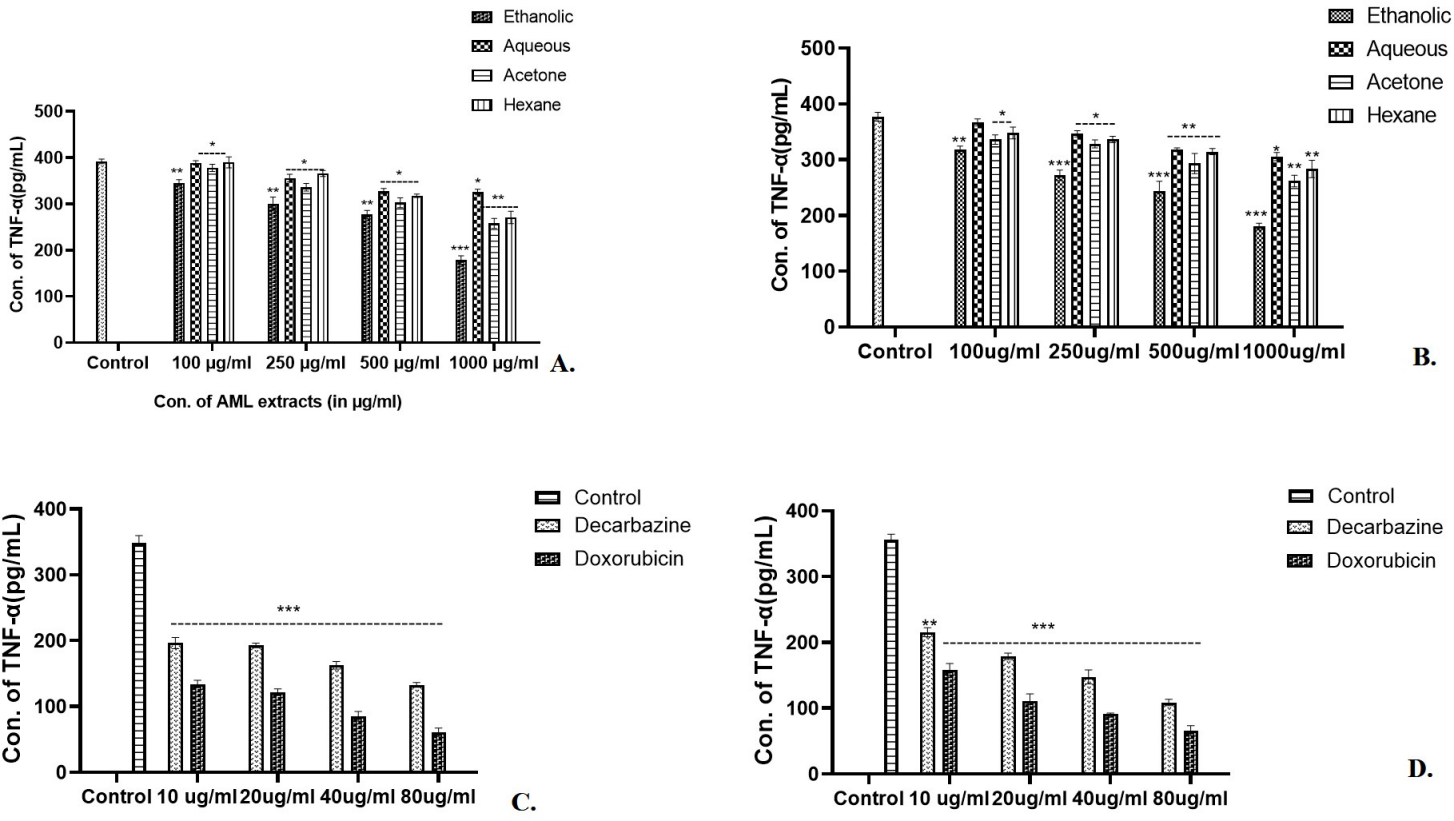
After exposure to the *Argemone mexicana* leaf extract, the expression of TNF-α cytokine was reduced. Eth AML extract (1,000 µg/ml) significantly reduced the expression of TNF-α in skin and colon cancer cell lines [*p*<0.001, **(A, B)**]. Using 80 µg/ml of doxorubicin and dacarbazine, TNF-α levels in skin cancer cells in **(C)** and colon cancer cells in **(D)** were considerably decreased. *** stands for *p<0.001*, ** for *p<0.01*, and * for *p<0.05* respectively. The two-way ANOVA followed by the Dunnett test was employed to calculate the significance.

In the case of skin cancer and colon cancer cell lines (**Figures 7A, B**), a highly significant (*p*<0.001) scaling down in concentration of TNF-α was observed by 1,000 µg/ml of ethanolic extract. The other extracts reduced the TNF-α concentrations of Aq (*p*<0.05), Ac (*p*<0.01), and Hex (*p*<0.01) at 1,000 µg/ml of AML extract (**Figures 7A, B**). A significant reduction by 80 µg/ml of standard chemotherapeutic drug dacarbazine (*p*<0.001) and doxorubicin (*p*<0.001) was also observed (**Figures 7C, D**) in both the cell lines. In conclusion, 1,000 µg/ml of ethanolic extract had the lowest TNF-α concentration (187.67 pg/ml) as compared with other extracts in the case of skin cancer cell lines. On the other hand, the AML extracts showed inhibition in TNF-α significantly by 1,000 µg/ml of ethanolic and acetone extracts (*p*<0.001) for colon cancer (**Figure 7B**), *p*<0.01 for acetone and hexane, and *p*<0.05 for aqueous extract.

### 3.6 Regulation of NF-kB pathway in the skin and colon cancer cell lines

**Figure 8:**
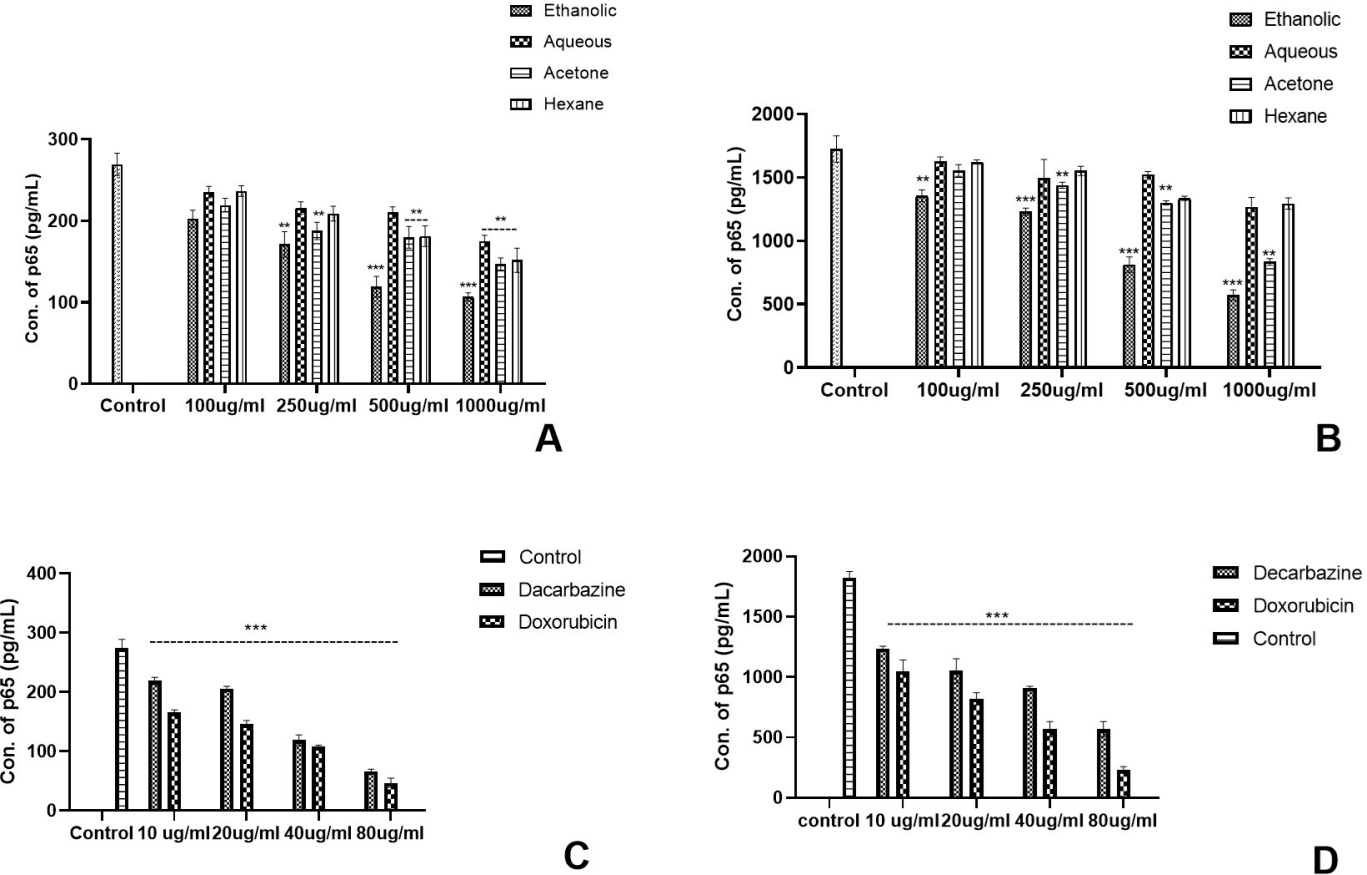
Effect of *Argemone mexicana* extracts on the reduction of the p65 transcription factor concentration in the skin cancer cell line. **(A)** presents the unstimulated cell culture where the amount of NF-kB has been significantly reduced under the influence of 1,000 µg/ml of ethanolic extract (*p<*0.001). **(B)** presents the stimulated cell culture where the amount of NF-kB expressed is higher in comparison with image **(A)**. The expression of the transcription factor significantly reduced *p<*0.001 with 1,000 µg/ml of ethanolic extract. **(C)** depicts the unstimulated cancer cell culture treated with the standard chemotherapeutic drugs dacarbazine and doxorubicin, while **(D)** represents the TNF-α-stimulated culture in the presence of dacarbazine and doxorubicin. A statistically significant (*p<*0.00*1*) reduction was observed with both the drugs. *** stands for *p<*0.001, ** for *p<*0.01, and * for *p<*0.05. Two-way ANOVA followed by the Dunnett test was employed to calculate the significance.

**Figure 9:**
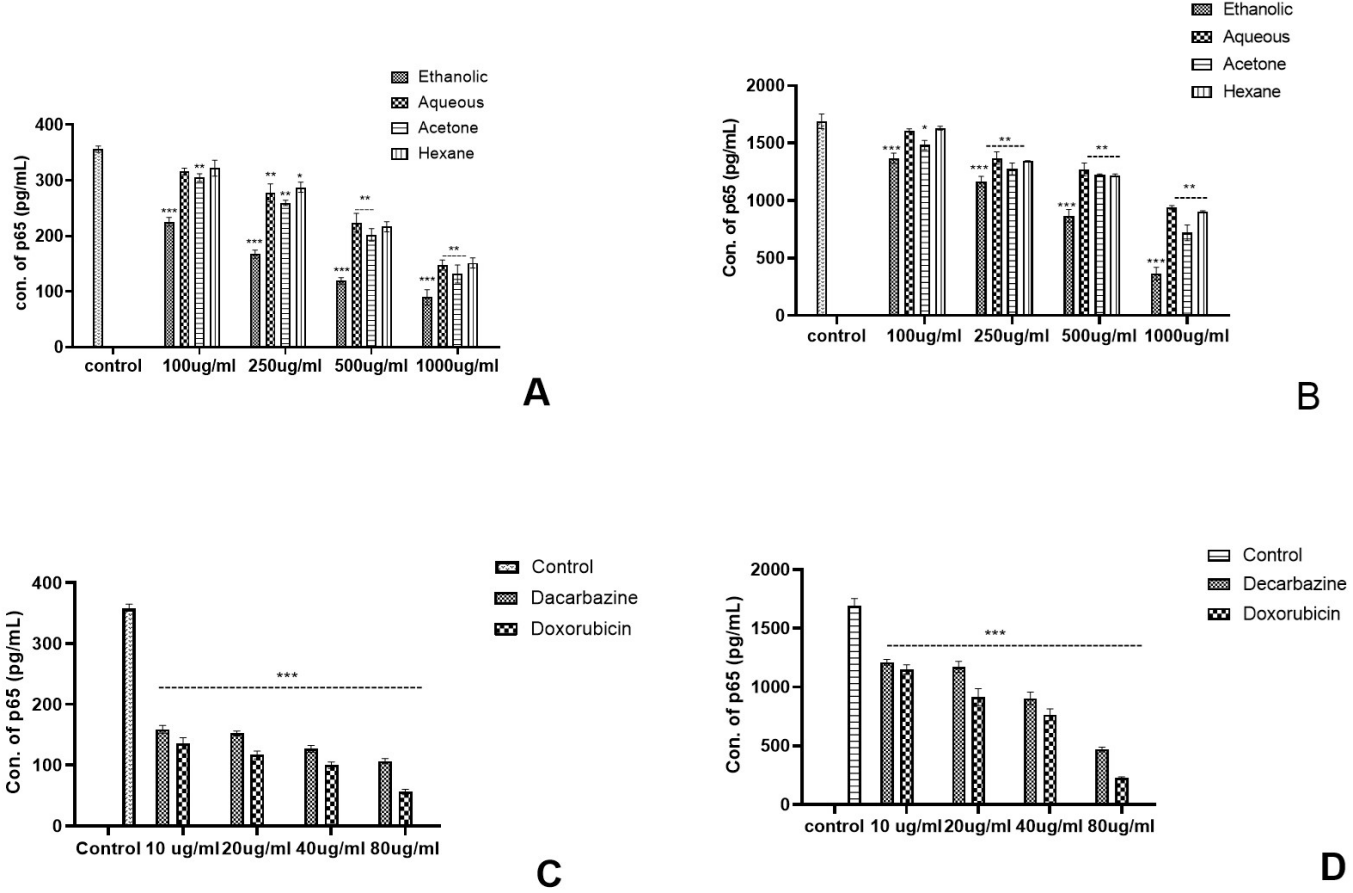
Effect of *Argemone mexicana* extracts on the reduction of the p65 transcription factor concentration in the colon cancer cell line. **(A)** represents the TNF-α unstimulated colon cancer cell culture where the concentration of *p*65 was significantly reduced by ethanolic extract at different doses while the highest dose 1,000 µg/ml reduced the *p*65 in other extracts. **(B)** represents the TNF-αstimulated cell culture where all the extracts significantly reduced the *p*65 concentration. However, maximum reduction was with the 1,000-µg/ml ethanolic extract. **(C)** summarizes the TNF-αunstimulated cancer cell culture treated with the standard chemotherapeutic drugs dacarbazine and doxorubicin. **(D)** represents the TNF-α-stimulated cancer cells treated with the same chemotherapeutic drugs with the same doses. Both the drugs statistically significantly (*p<0.001*) reduced the *p*65 concentration. *** stands for *p<0.001*, ** for *p<0.01*, and * for *p<0.05*. Two-way ANOVA followed by the Dunnett test was employed to calculate the significance.

The experiment was conducted as a comparative study to differentiate the activation of the NF-kB pathway by analysing the concentration of the p65 transcription factor in TNF-α stimulated and unstimulated cancer cell cultures as per the protocol by Ernst et al. It was evaluated that p65 in the TNF-α-stimulated cell culture was ~8–9 times higher than the unstimulated cells. In skin cancer, the concentration of p65 transcription factor was significantly reduced in unstimulated cancer cells by Eth extract of Argemone mexicana Linn (**Figures 8A, C**) and stimulated culture (**Figures 8B, D**). However, less significant reduction in p65 concentration was observed with the aqueous and hexane extracts of *Argemone mexicana* Linn leaves in skin as well as colon cancer cell lines (**Figures 9A, B**). A 80-µg/ml concentration of standard chemotherapeutic drug was found to significantly reduce the p65 subunit by Da (52.36%) and Do (78.56%) (**Figure 8C**). On the other hand, in the unstimulated cells, the p65 concentration was extremely reduced in the ethanolic extract from 268.18 pg/ml to 112.89 pg/ml and significantly reduced to 145.56 pg/ml in the acetone extract, while in aqueous and hexane extracts, no significant reduction was observed.

In comparison with skin cancer cell lines in colon cancer cell lines, significant reduction was noted with all the extracts in TNF-α-stimulated as well as unstimulated cultures. However, the highest reduction was observed with ethanolic extracts at different concentrations as compared with the respective concentrations of the other extracts (**Figures 9A, B**). Both the standard chemotherapeutic drugs dacarbazine and doxorubicin reduced the concentration p65 transcription factor. Doxorubicin reduced the concentration of p65 from 355.12 pg/ml to 45.16 pg/ml in TNF-α-unstimulated and from 1782.79 pg/ml to 228.16 pg/ml values in stimulated cultures, respectively (**Figures 9C, D**). The ethanolic AML extract was found to reduce the p65 concentration from 355.57 pg/ml to 88.13 pg/ml in the unstimulated cell culture.

## 4 Discussion

Cancer, the second-biggest cause of mortality after heart disease, is a global health issue. In recent years, interest in natural compounds having anticancer therapeutic potential has grown. Without a doubt, pharmacological studies of plant extracts as a source of secondary metabolites (23) are the most crucial step in discovering molecules from plants that could potentially serve as medicines. *Argemone mexicana* Linn is one of the plants known for its anticancer activity. Indians use different parts of the plant to treat a variety of diseases, including jaundice, scabies, fungal infections, ulcers, asthma, intestinal infections, skin, cough, and other conditions (24, 25). The plants’ pooled phytochemicals and their derivatives, such as sterols, flavonoids, and alkaloids, could potentially treat cancer (26).

We performed the LC/MS analysis for four distinct leaf extracts (ethanolic, aqueous, acetone, and hexane) and predicted the compounds based on their molecular weights. The study predicted various types of alkaloids and flavonoids, such as berberine, oleandrigenin-ß-D-digi (neritaloside), and pancorine, among others. All the extracts shared octadecanoic acid (M.W. 402 Da). This was also found in the GC/MS analysis done by Khan and Bhaduria in 2019 (27). In addition to this, oleandrigenin-ß-D-digi (Neritaloside) (592 Da) was found in all three extracts. This substance has been found in *Nerium oleander* and has been studied extensively for its ability to fight cancer by various researchers (28). The literature has already reported the anticancer properties of all the identified compounds (29, 30).

It was observed that the compounds allocryptopine, oxyberberine, chelerythrine, linoleic acid, and neritaloside, common in all extracts, support the experimentally observed effects. It was found that the PI3K-Akt signalling pathway is very important for controlling inflammation and tumour growth. The effect of this pathway is by modulating the levels of pro-inflammatory cytokines like TNF-α and the activity of transcription factors like NF-kB-p65 (31, 32). The results of the tests with *Argemone mexicana* Linn extracts show that natural compounds may be able to target these pathways to help treat cancer and inflammatory diseases.

Despite being a well-known toxic plant, sanguinarine has garnered significant attention in the past, particularly following the case of dropsy, which resulted from the mixing of plant seeds with mustard seeds due to their similarity (33), However, a study conducted in Mali, South Africa, revealed the effectiveness of using plant extract as a treatment for malaria. The study was conducted in clinical trials in Mali and Switzerland (10). These showed that the other parts are safe because they do not contain sanguinarine. This led to our study, in which we tested the MNTD of *Argemone mexicana* Linn leaf extracts on fibroblast cell lines (L929). It was evaluated that the dose of 2,000 µg/ml of all the extracts was found to be toxic and showed inhibition in proliferation of fibroblast cells, and the MNTD was determined to be 1,000 µg/ml of all the extracts, which were used for further study.

The study used skin cancer cell lines (A-431) and colon cancer cell lines (COLO 320 DM) to determine the anti-cancerous activity. In this experiment, 1,000 µg/ml of the ethanolic extract showed the highest inhibition percentage for both cell lines compared with other extracts.

Prabhakaran et al. (34) cited research on *Argemone mexicana.* Linn flowers’ anticancer effect against HEPG2 human liver cancer cells using alkaloids such as angoline (34). Other *in vitro* studies reported the activity of compounds like chelerythrine on the human nasopharyngeal carcinoma cell line (HONE-1) and the human gastric cell line (NUGC) (35). The LC/MS analysis of the ethanolic AML extract had predicted the presence of a chelerythrine component (ref. **Table 1**), which may be consistent with the extract’s potent anticancer effects. Gacche et al. (36) conducted a study to examine the cytotoxic effects of three different AML extracts. The results showed that the ethanolic extract had the most favourable impact. According to the reports, *Argemone mexicana* Linn leaves were found to exhibit cytotoxic effects on a variety of cell lines, including those representing metastatic breast cancer (MDA-MB-435S), colon cancer (HT29), gastric cancer (AGS), cervical cancer (HELA), leukaemia (HL-60), and renal carcinoma (PN-15) (37, 38). Singh et al. (39) found 200 µg/ml of isolated alkaloids effective in colon cancer cell lines. This study tested the crude extract on cell lines, and the synergistic effect of the phytoconstituents confirmed their activity and high dose compared with isolated chemicals (40). The findings on cancer cell growth inhibition by ethanolic AML extract were supported by a dose-dependent downregulation in the expression of the pro-inflammatory cytokine TNF-α. In the test, 1,000 µg/ml of the ethanolic extract was shown to significantly lower the level of TNF-α in skin cancer (*p*<0.001 at 187.67 pg/ml) and colon cancer (*p*<0.001 at 175.99 pg/ml) cell lines (b). The results could be correlated with the study by Monterrosas-Brisson et al. (41), where it was concluded that the methanolic extract of *Argemone mexicana* Linn lowered TNF-α in inflammation in an ear oedema-induced mouse model.

The nuclear factor kappa B (NF-kB) family of transcription factors plays a key role in inflammation and cancer. Various biological stimuli activate the cytoplasmic NF-kB protein. Two different pathways, conventional and non-conventional, turn on NF-kB. These pathways involve complex molecular interactions involving adaptor proteins, phosphorylation, and ubiquitinase enzymes. The nucleus experiences an increase in NF-kB translocation, which in turn regulates gene expression (42). In cancer, NF-kB activation is associated with cell proliferation, survival, invasion, and angiogenesis, making it a viable therapeutic target. Inhibitors of NF-kB have been found to suppress tumour growth and induce apoptosis in malignant cells (43). The NF-kB transcription factor was similarly tapped in our study to understand the potential of AML as an anticancer agent. It was found that the concentration of p65 (the transcription factor of the NF-kB pathway) was found to be reduced in TNF-α-stimulated skin cancer cells (232.89 pg/ml), colon cancer cells (325.89 pg/ml), unstimulated skin cancer cells (63.16 pg/ml), and colon cancer cell lines (88.13 pg/ml) as compared with control cultures by the AML extract. The ethanolic extract exhibited a significant reduction (*p*> 0.001) at 1000 µg/ml in the cell cultures.

The downregulation of TNF-α expression and the modulation of NF-kB-p65 transcription factor in a dose-dependent manner, as observed in the experiments, can be correlated with the involvement of *Argemone mexicana* phytochemicals on PI3K-Akt, as concluded in the network pharmacology study (ref. **Figure 2**). There is various evidence indicating a connection between the PI3K-Akt signalling pathway and NF-kB (32, 44, 45). Despite not being a part of the main PI3/AKT pathway, NF-kB is located downstream of AKT (22). AKT activation plays a critical role in several NF-kB tumorigenic activities (46). This downregulation is significant in cases where excessive TNF-α production contributes to disease pathology, such as skin cancer and colon cancer. It can influence NF-kB activity by regulating its nuclear translocation and DNA binding ability. In the context of inflammation and cancer, aberrant NF-kB activation can lead to the upregulation of pro-inflammatory genes and promote tumorigenesis. So, changing the activity of NF-kB through the PI3K-Akt pathway can help control inflammatory responses and stop the growth of tumours. The ethanolic extracts of *Argemone mexicana* Linn have been found to exhibit anti-inflammatory properties by downregulating TNF-α expression and modulating NF-kB-p65 transcription factor activity, suggesting their potential therapeutic efficacy in inflammatory and cancer-related conditions.

## 5 Conclusion

The present study evaluated the anticancer properties of *Argemone mexicana* Linn, which is a plant already known for its anti-inflammatory and other medicinal properties. Different types of extracts (Eth, Aq, Ac, and Hex) in different doses were tested with A431 and COLO 320DM cell lines, and a significant inhibition in cell proliferation could be seen in a dose-dependent manner. The observed inhibition could be validated by studying the TNF-α expression and regulation of the NF-kB-p65 transcription factor and its signalling pathway. The latter are well known to play a role in inflammatory diseases, including cancer. The 1,000 µg/ml of ethanolic extract was found to be the most effective in the experiments conducted. We can conclude that the plant possesses anticancer properties, and further experimental setups, such as *in vivo* and other preclinical studies, could further explore the medicinal potential of *Argemone mexicana* in cancer treatment.

## Data Availability

The original contributions presented in the study are included in the article/**Supplementary Material**. Further inquiries can be directed to the corresponding author.
