# Supplementary material for: Metabolomics and network pharmacology–guided analysis of TNF-α expression by Argemone mexicana (Linn) targeting NF-kB the signalling pathway in cancer cell lines
Source: Front Oncol. 2024 Dec 2;14:1502819. doi: 10.3389/fonc.2024.1502819 (PMC11648424; doi:10.3389/fonc.2024.1502819)
Supplement: Supplementary file 1 [file Table1.docx]

Supplementary Material

**Supplemental Data**

**Table S1: LC parameters in LC/MS analysis**

|  | **A multi-step linear-gradient qualitative analysis.** | | | |
| --- | --- | --- | --- | --- |
| **S. No.** | **Time** | **Flow rate**  **(mL/min)** | **Solvent A**  **(Acetonitrile)** | **Solvent B (0.1% Formic acid in 95:5 v/v water/ acetonitrile)** |
|  | 0.1 | 0.25 | 5 | 95 |
|  | 1.0 | 0.25 | 5 | 95 |
|  | 10.0 | 0.25 | 30 | 70 |
|  | 14.0 | 0.25 | 60 | 40 |
|  | 16.0 | 0.25 | 60 | 40 |
|  | 24.0 | 0.25 | 80 | 20 |
|  | 32.0 | 0.25 | 80 | 20 |
|  | 35.0 | 0.25 | 5 | 95 |
|  | 40.0 | 0.25 | 5 | 95 |

**Table S2: MS Parameters in LC/MS analysis:**

|  | | **Table-2 ESI parameters** | |
| --- | --- | --- | --- |
|  | Source Temperature | | 120°C |
|  | Desolvation Temperature | | 350°C |
|  | Capillary | | 3.5(kV) |
|  | Cone | | 30V |
|  | Cone Gas Flow | | 50 (L/Hr) |
|  | Desolvation Gas Flow | | 950 (L/Hr) |

**Table S3. LC/MS analysis of AML extracts**

**Table S3.1:** Compounds from Ethanolic extract LC/MS analysis of AML ethanolic extract along with their molecular weight (Da) , predicted compound and reported biological activities

| **S. No.** | **Rt. (min)** | **MW(Da)** | **ES+** | | **Compound Name** | **Biological activity** |
| --- | --- | --- | --- | --- | --- | --- |
|  |  |  | **[M+H]^+^** | **[M+Na]^+^** |  |  |
| **1.** | 7.47 | 282.55 | 283 |  | Oleic acid/  Octadecenoic acid | Antioxidant, Antimicrobial, Anti-cancerous, Anti-inflammatory, Insecticidal [1] |
| **2.** | 7.98 | 208.2 | 209 | 340 | Pentadecyne | Anti-oxidant, Anti-microbial [2] |
| **3.** | 8.87 | 196.2 | 197 | 214 | Argenaxine | Cytotoxic [3] |
| **4.** | 8.91 | 353.4 | 354 |  | protomexicine | Colon cancer [4] |
| **5.** | 9.50 | 369.14 | 370 |  | Allocryptopine | Antimalarial [5] |
| **6.** | 9.77 | 337.13 | 338 |  | Jatrorrhizine | Colon cancer [6] |
| **7.** | 10.39 | 351.4 | 354 |  | oxyberberine | Anti cancerous (Colon), Antifungal [7] |
| **8.** | 11.04 | 335.12 | 336 |  | Berberine | Cervical cancer, ovarian cancer, breast cancer, lung cancer, stomach cancer, leukaemia, and B-cell lymphoma [8] |
| **9** | 13.04 | 348.12 | 346 |  | Chelerythrine | Cytotoxic, Anticanerous (Gastric, nasopharyngeal and breast)  [9] |
| **10** | 18.77 | 333.4 | 334 |  | Pancorine | Gall bladder cancer, breast cancer [10] |
| **11** | 18.81 | 338.13 | 339 |  | Dehydrocorydalmine | Anti-cancerous [11] |
| **12** | 20.64 | 279.3 | 280.3 | 301 | 13-oxoprotopine/  Linoleic Acid | Anti fertility, effect on guinea pig ileum, Mollucicidal [12] |
| **13** | 27.31 | 592.9 | 594 |  | Oleandrigenin-ß-D-digi (Neritaloside) | leprosy, malaria, ringworm, anti-cancerous activity [13] |

**Table S.3.2:** LC/MS analysis of AML aqueous extract along with molecular weight (Da), predicted compound and reported biological activities

| **S. No.** | **Rt. (min)** | **MW(Da)** | **ES+** | | **Compound Name** | **Biological activity** |
| --- | --- | --- | --- | --- | --- | --- |
|  |  |  | **[M+H]^+^** | **[M+Na]^+^** |  |  |
| **1.** | 9.01 | 353.4 | 354 |  | protomexicine | Anti-Cancer [4] |
| **2.** | 9.60 | 369.14 | 370 |  | Allocryptopine | Antimalarial and anti-cancerous [5] |
| **3.** | 20.6 | 279.3 | 279 |  | 13-oxoprotopine | Anti fertility, effect on guinea pig ileum, , Mollucicidal [12] |
| **4.** | 23.3 | 282.55 | 286 |  | Octadecanoic acid | Antioxidant, Antimicrobial, Anti-cancerous, Anti-inflammatory, Insecticidal [1] |

**Table S.3.3:** LC/MS analysis of AML acetone extract along with molecular weight (Da), predicted compound and reported biological activities

| **S. No.** | **Rt. (min)** | **MW(Da)** | **ES+** | | **Compound Name** | **Biological activity** |
| --- | --- | --- | --- | --- | --- | --- |
|  |  |  | **[M+H]^+^** | **[M+Na]^+^** |  |  |
| **1.** | 7.79 | 339.1 | 340 |  | Reticuline | Cytotoxic, Anti cancerous (nasopharyngeal, Gastric) [9] |
| **2.** | 8.24 | 196.2 | 197 | 214 | Argenaxine | Cytotoxic [3] |
| **3.** | 8.89 | 353.4 | 354 |  | protomexicine | Colon cancer [6] |
| **4.** | 9.46 | 369.14 | 370 |  | Dihydrochelerythrine/  Allocryptopine | Antimalarial and nti-cancerous [5] |
| **5.** | 9.70 | 337.13 | 338 |  | Jatrorrhizine | Colon cancer [6] |
| **6.** | 10.32 | 353.4 | 354 |  | protomexicine | Leukemia, stomach cancer, ovarian cancer, breast cancer, lung cancer, B-cell lymphoma, and cervical cancer [4] |
| **7.** | 10.98 | 335.12 | 336 |  | Berberine | Gall bladder cancer, breast cancer [8] |
| **8.** | 18.74 | 333.4 | 334 |  | Pancorine | leprosy, malaria, ringworm, anti-cancerous activity [10] |
| **9.** | 27.27 | 592.9 | 593 |  | Oleandrigenin-ß-D-digi (Neritaloside) | leprosy, malaria, ringworm, anti-cancerous activity [13] |

**Table S.3.4:** LC/MS analysis of AML hexane extract along molecular weight (Da), predicted compound and reported biological activities

| **S. No.** | **Rt. (min)** | **MW(Da)** | **ES+** | | **Compound Name** | **Biological activity** |
| --- | --- | --- | --- | --- | --- | --- |
|  |  |  | **[M+H]^+^** | **[M+Na]^+^** |  |  |
| **1.** | 7.36 | 379.8 | 380 |  | Linoleic Acid | Anti fertility, effect on guinea pig ileum, , Mollucicidal, antibacterial, anticancer [12] |
| **2.** | 7.90 | 321.8 | 322 |  | Arachidic Acid | Anti-inflammatory [14] |
| **3.** | 8.05 | 341.1 | 342 |  | Magnoflorine | Anti-cancer, anti-inflammatory (Xu *et al.,*  2020) |
| **4.** | 8.29 | 196.2 | 194 | 214 | Argenaxine | Cytotoxic [3] |
| **5.** | 8.84 | 353.4 | 354 |  | protomexicine | Leukemia, stomach cancer, ovarian cancer, breast cancer, lung cancer, B-cell lymphoma, and cervical cancer [15] |
| **6.** | 9.46 | 369.14 | 370 |  | Allocryptopine | Antimalarial [5] |
| **7.** | 10.90 | 335.12 | 376 |  | Berberine | Breast cancer, lung cancer, stomach cancer, ovarian cancer, leukaemia, B-cell lymphoma, cervical cancer, and ovarian cancer [8] |
| **8.** | 17.33 | 276.5 | 277 |  | Argemonic Acid | Microbial infection, skin disease, rheumatoid arthritis [3] |
| **9.** | 20.36 | 333.4 | 334 |  | Pancorine | Anti-cancerous [10] |
| **10.** | 24.45 | 279.3 | 279 |  | 13-oxoprotopine | Anti fertility, effect on guinea pig ileum, Mollucicidal [12] |
| **11.** | 27.27 | 592.9 | 593 |  | Oleandrigenin-ß-D-digi (Neritaloside) | leprosy, malaria, ringworm, anti-cancerous activity [13] |

**References**

1. Alabi, K. A., Lajide, L., & Owolabi, B. J. (2018). Biological activity of oleic acid and its primary amide: Experimental and Computational studies. *Journal of Chemical Society of Nigeria*, *43*(2).
2. Lazreg-Aref, H., Mars, M., Fekih, A., Aouni, M., & Said, K. (2012). Chemical composition and antibacterial activity of a hexane extract of Tunisian caprifig latex from the unripe fruit of Ficus carica. *Pharmaceutical Biology*, *50*(4), 407-412.
3. Brahmachari, G., Gorai, D., & Roy, R. (2013). Argemone mexicana: chemical and pharmacological aspects. *Revista Brasileira de Farmacognosia*, *23*, 559-567.
4. More, N. V. and Kharat A. S. (2016) Antifungal and Anticancer Potential of *Argemone mexicana* L. *Medicines*. 28: 1-10.
5. Dong, Z., Wang, Y. H., Tang, Z. S., Li, C. H., Jiang, T., Yang, Z. H., & Zeng, J. G. (2022). Exploring the Anti-inflammatory Effects of Protopine Total Alkaloids of Macleaya Cordata (Willd.) R. Br. *Frontiers in Veterinary Science*, *9*, 935201.
6. Singh, S.; Verma, M.; Malhotra, M.; Prakash, S.; & Singh, T. D. Cytotoxicity of alkaloids isolated from *Argemone mexicana* on SW480 human colon cancer cell line. *Pharmaceutical biology* 2016, *54*(4), 740-745.
7. Anwar, M. A., Tabassam, S., Gulfraz, M., Sheeraz Ahmad, M., Raja, G. K., & Arshad, M. (2020). Isolation of oxyberberine and β-sitosterol from Berberis lycium Royle root bark extract and in vitro cytotoxicity against liver and lung cancer cell lines. *Evidence-Based Complementary and Alternative Medicine*, *2020*.
8. Och, A., Podgórski, R., & Nowak, R. (2020). Biological activity of berberine—a summary update. *Toxins*, *12*(11), 713.
9. Valipour, M., Zarghi, A., Ebrahimzadeh, M. A., & Irannejad, H. (2021). Therapeutic potential of chelerythrine as a multi-purpose adjuvant for the treatment of COVID-19. *Cell Cycle*, *20*(22), 2321-2336.
10. Iqbal, J.; Abbasi, B. A.; Mahmood, T.; Kanwal, S.; Ali, B.; Shah, S. A. & Khalil, A. T. Plant-derived anticancer agents: A green anticancer approach. *Asian Pac. J. Trop. Biome*d 2017, *7(12),* 1129-1150.
11. Miret-Casals, L., Baelo, A., Julián, E., Astola, J., Lobo-Ruiz, A., Albericio, F., & Torrents, E. (2018). Hydroxylamine derivatives as a new paradigm in the search of antibacterial agents. *ACS omega*, *3*(12), 17057-17069.
12. Wangchuk, P., Keller, P. A., Pyne, S. G., Sastraruji, T., Taweechotipatr, M., Rattanajak, R., ... & Kamchonwongpaisan, S. (2012). Phytochemical and biological activity studies of the Bhutanese medicinal plant Corydalis crispa. *Natural Product Communications*, *7*(5), 1934578X1200700507.
13. Zhai, J., & Dong, X. (2022). Oleandrin: a systematic review of its natural sources, structural properties, detection methods, pharmacokinetics and toxicology. *Frontiers in Pharmacology*, *13*, 822726.
14. Brash, A. R. (2001). Arachidonic acid as a bioactive molecule. *The Journal of clinical investigation*, *107*(11), 1339-1345.
15. Chang, Y. C., F. R Chang, A. T Khalil, P. W Hsieh and Y. C Wu (2003) Cytotoxic benzophenanthridine and benzylisoquinoline alkaloids from *Argemone mexicana. Z. Naturforsc*h. 58: 521-526.
